# Reliability of measuring pectoralis minor muscle resting length in subjects with and without signs of shoulder impingement

**DOI:** 10.1590/bjpt-rbf.2014.0146

**Published:** 2016-03-15

**Authors:** Dayana P. Rosa, John D. Borstad, Elisa D. Pires, Paula R. Camargo

**Affiliations:** 1 Programa de Pós-gradução em Fisioterapia, Universidade Metodista de Piracicaba (UNIMEP), Piracicaba, SP, Brazil; 2 Department of Physical Therapy, The Ohio State University (OSU), Columbus, OH, USA; 3 Departamento de Fisioterapia, Universidade Federal de São Carlos (UFSCar), São Carlos, SP, Brazil

**Keywords:** measurement, scapula, physical therapy, tightness

## Abstract

**Background:**

Pectoralis minor adaptive shortening may change scapula resting position and scapular kinematics during arm elevation. A reliable and clinically feasible method for measuring pectoralis minor length will be useful for clinical decision making when evaluating and treating individuals with shoulder pain and dysfunction.

**Objectives:**

To evaluate intrarater, interrater, and between-day reliability of a pectoralis minor (PM) muscle length measurement in subjects with and without signs of shoulder impingement.

**Method:**

A convenience sample of 100 individuals (50 asymptomatic and 50 symptomatic) participated in this study. Intra- and interrater reliability of the measurement was estimated in 50 individuals (25 asymptomatic and 25 symptomatic), and between-day reliability of the measurement repeated over an interval of 7 days was estimated in an independent sample of 50 additional participants. Pectoralis minor length was measured using a flexible tape measure with subjects standing.

**Results:**

Intraclass correlation coefficients (ICC_3,k_) for intrarater and interrater reliability ranged from 0.86-0.97 and 0.95 for between-day reliability in both groups. Standard error of measurements (SEM) ranged from 0.30-0.42 cm, 0.70-0.84 cm, and 0.40-0.41 cm for intrarater, interrater, and between-day reliability, respectively, across the sample. The minimal detectable change (MDC) for between-day measurements ranged from 1.13-1.14 cm for both groups.

**Conclusions:**

In asymptomatic individuals and in those with signs of shoulder impingement, a single rater or pair of raters can measure pectoralis minor muscle length using a tape measure with very good reliability. This measurement can also be reliably used by the same rater over a seven day interval.

## BULLET POINTS

- Pectoralis Minor length can be reliably measured using a tape measure.
- The presence of shoulder pain does not decrease reliability.
- Reliability is very good for measurements taken over a seven-day interval.
- Reliability estimates should not be generalized to different shoulder conditions.

## Introduction

The pectoralis minor (PM) muscle attaches to the coracoid process of the scapula and inserts on ribs three, four, and five near the costosternal junction. It is the only scapulothoracic muscle with an anterior thoracic attachment^1^^,^ 2. It has been theorized that a habitual forward shoulder posture causes an adaptive length decrease of the pectoralis minor, which may subsequently contribute to movement alterations and/or shoulder pain^3^^-^ 5. Repetitive use of the upper extremity for activities that protract and downwardly rotate the scapula may also contribute to adaptive shortening^6^^,^ 7. The muscles' orientation determines that it will produce scapular downward rotation, anterior tilt, and internal rotation when it activates, and it is therefore an antagonist to upward rotation, posterior tilt, and external rotation, which are considered to be normal during arm elevation^8^. In support of this construct, pectoralis minor adaptive shortening has been associated with changes in the resting position of the scapula^3^ and altered scapular kinematics during arm elevation^1^. Specifically, a group of asymptomatic subjects with relatively short pectoralis minor muscle resting length had decreased scapular posterior tilting and external rotation during arm elevation when compared to those with a relatively long muscle resting length^1^. With PM resting length identified as a potential contributor to detrimental shoulder kinematics, a reliable clinical assessment of resting length will be valuable for clinicians as they plan interventions and assess the effect of those interventions.

Measuring PM muscle length with an electromagnetic motion capture system using the coracoid process and the fourth rib as origin-insertion landmarks has shown excellent validity^9^ and is considered the "gold standard" method. However, the electromagnetic system is time-consuming, expensive, not typically available to clinicians, and mainly used for research purposes. Thus, there is a need for a more clinically feasible instrument to assess PM length in subjects with postural deviations or shoulder dysfunction^3^. A tape measure and caliper both demonstrated good reliability with the electromagnetic system (Intraclass Correlation Coefficient - ICC_3,1_ ranging from 0.82 0.87) to measure pectoralis minor muscle length within the same day by the same rater^1^^,^ 9. Although both tools had good reliability with the electromagnetic system, a tape measure is more readily available and easily manipulated in clinical practice.

Struyf et al.^10^ recently used a caliper to measure the length of the pectoralis minor and demonstrated excellent (ICC_2,1_ ranging from 0.87-0.93) and good (ICC_2,1_ ranging from 0.76-0.87) intrarater reliability when reporting the Pectoralis Minor Index (PMI) in subjects with shoulder pain and asymptomatic subjects, respectively^10^. Moderate interrater reliability was demonstrated in both groups (ICC_2,1_ ranging from 0.64-0.72). The PMI used by Struyf et al.^10^ normalizes resting PM length to subject height^1^, which is therefore not a direct assessment of absolute measurement reliability. The PMI was first proposed by Borstad and Ludewig^1^ to classify people into relatively short and long PM groups and evaluate the effect of PM length on scapula kinematics. However, for assessing an individual patient and to evaluate the effectiveness of interventions to lengthen PM, a direct measurement of the muscle is more clinically relevant. In addition, as there are currently no normative values of the PMI reported in the literature, a direct measurement is more useful for making clinical decisions about an individual patient.

Another important variable that is missing in the literature is a reliability estimate of measuring PM length over time. This is a critical research gap because it limits a clinician's knowledge of how consistent the PM measurement is when used in the same patient over the course of their intervention program. Between-day reliability estimates are needed to provide clinicians with the ability to assess pectoralis minor length change over time due to treatment effects or other influences such as work or postural habits. These between-day reliability estimates are even more valuable if they have assessed a time interval that represents how the measurement will typically be used in the clinic.

The purpose of this study was to evaluate the intrarater, interrater, and between-day reliability of using a tape measure to assess pectoralis minor resting length in asymptomatic individuals and individuals with signs of shoulder impingement. Measurements of agreement, such as the minimal detectable change (MDC), standard error of measurement (SEM) of the measurement, and Bland Altman plots, were also determined in order to facilitate clinical interpretation of change over time.

## Method

A convenience sample of 100 individuals (50 asymptomatic and 50 symptomatic) participated in this study. Individuals were recruited by means of fliers and direct contact from a local university setting and the community. Symptomatic subjects were recruited from a physical therapy waiting list at the clinic of Universidade Federal de São Carlos, (UFSCar), São Carlos, SP, Brazil and orthopedic clinics. All subjects were screened for eligibility by the first author and were required to be between 18 and 35 years of age. Asymptomatic individuals were included if they had no history of shoulder or cervical pathology. Because of the proposed relationship between pectoralis minor length, scapula kinematic alterations, and subacromial impingement syndrome^1^^,^ 3, individuals with signs and symptoms consistent with impingement were targeted for inclusion.

The diagnosis for shoulder impingement was based on a clinical examination and self-reported history. To be classified as having shoulder impingement, subjects had to present with at least three^7^^,^ 11 ^-^ 16 of the following: positive Neer^17^ test, positive Hawkins test, positive Jobe and Moynes^18^ test, pain with passive or isometric resisted shoulder lateral rotation^8^^,^ 19, pain with active shoulder elevation^20^, pain with palpation of rotator cuff tendons, and anterolateral shoulder pain. Individuals were excluded if they were pregnant; had ligamentous laxity based on positive Sulcus test^21^; had apprehension during Apprehension test^22^; had history of clavicle, scapula, or humerus fracture; had systemic illnesses; or had received any treatment for shoulder pain in the last 6 months.

The study was approved by the Ethics Committee of the Universidade Metodista de Piracicaba (UNIMEP), Piracicaba, SP, Brazil (protocol number 100/12). All subjects gave their written and informed consent to participate in this study, which was conducted according to the Declaration of Helsinki.

Only the symptomatic shoulder was evaluated in the symptomatic individuals. For the asymptomatic participants, the side evaluated was randomly determined with a randomization list created by a computer program. Half of the sample (25 asymptomatic and 25 symptomatic; Sample 1) was used to determine intra- and interrater reliability, while the other half (Sample 2) was used to determine between-day reliability. Two independent samples were used to reduce the potential for bias that may have occurred with multiple measurements, such as postural adjustments by subjects or familiarity with subjects' previous measurements by raters.

Pectoralis minor resting length was measured with a tape measure with 0.10 cm resolution. To determine the interrater reliability for sample 1, all measurements were taken by two investigators who were blinded to all measures. To prevent bias, one side of the tape measure was covered with adhesive tape, blinding the examiners to the value measured. A third examiner read and recorded the measurements. To standardize the measurement technique, the primary and secondary investigators underwent training, which included studying relevant anatomy, systematically locating and palpating the coracoid process and the fourth rib landmarks, and then practicing all procedures on 10 healthy individuals. It is estimated that the training totaled approximately 6 hours.

**Figure 1.**
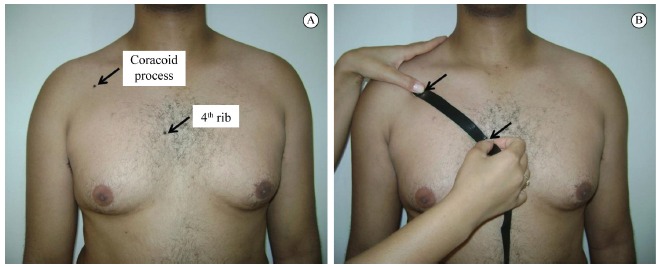
Bony landmarks used to represent pectoralis minor length (A) and measurement of pectoralis minor length taken with a tape measure (B).

Two bony landmarks representing the insertion and origin of the muscle were palpated, marked with a pencil, and used to represent pectoralis minor length: the caudal edge of the fourth rib at the sternum and the inferomedial aspect of the coracoid process (Figure 1A)^1^. The distance between these landmarks was estimated using the tape measure for two trials with two minutes between each trial. The caudal edge of the fourth rib was located by identifying the sternal part of the clavicle and counting down the intercostal spaces until fourth rib. The coracoid process landmark was located by palpating just distal to the concave region of the acromial end of the clavicle. During the measurements, participants were asked to remain in a standing and relaxed posture with their arms at their sides in a neutral position and to avoid postural correction (Figure 1B). Subjects were also instructed to exhale completely during the measure. The pencil marks were removed after each measurement. The same procedure was followed by the primary and secondary investigators. Following one evaluator's two measurements, subjects were allowed to rest comfortably for 5 minutes prior to the two measurements by the other evaluator. A randomization list was used to determine which evaluator measured first and second.

A test-retest design was used for sample 2 with one rater performing all measurements. Participants from sample 2 were measured in two sessions separated by an interval of seven days. This interval was selected to represent the timeframe over which the measurement is likely to be used by clinicians to assess change in PM length.

The data were analyzed using the SPSS statistical package (17.0 Version). Data were normally distributed (p>0.05) as verified by the Kolmogorov-Smirnov test. A one-way ANOVA (Analysis of Variance) and an independent *t*-test were used to determine if baseline differences existed between groups for demographic variables and duration of pain, respectively. The relative reliability was determined by calculating the ICC for intrarater (ICC_3,1_), between-day (ICC_3,2_) and interrater (ICC_3,2_) reliability^23^. The intrarater reliability was evaluated by separately comparing the two measurement trials from the first and second sessions. The interrater reliability was estimated using the mean of the two tape measure trials of each evaluator. The between day reliability was estimated by comparing the mean of the two trials from each session. The ICC values were considered poor when below 0.20; fair from 0.21 to 0.40; moderate from 0.41 to 0.60; good from 0.61 to 0.80; and very good from 0.81 to 1.00^24^. The absolute reliability was defined as the SEM and MDC using the following formulas:


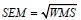
, where WMS (within mean square) is the within subjects mean square error term from a one-way ANOVA with subjects as the independent variable^25^; and 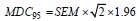, for 95% confidence interval (CI)^26^^,^ 27.

The SEM provides a value for measurement error for any given trial (intrarater reliability), any test occasion (between-day reliability), and any evaluator (interrater reliability)^25^^,^ 28. The MDC is an estimate of the smallest amount of change between repeated measures that can be considered to be a true change beyond measurement error^27^^-^ 29 The MDC represents an outer limit of the amount of random variation that 95% of stable subjects will demonstrate when measures are collected on separate occasions.

Bland-Altman plots^30^ were constructed to allow visual examination of the tape measure agreement between-days. The plots were constructed using MedCalc Software (Mariakerke, Belgium).

## Results

Table 1 shows no differences among the groups in the descriptive data (p>0.05).

**Table 1.** Descriptive data of the subjects.

	**Sample 1**	**Sample 2**	**p value**		
	**Asymptomatic (n=25)**	**Symptomatic (n=25)**	**Asymptomatic (n=25)**	**Symptomatic (n=25)**	
Age (years)*	25.72±3.52	25.52±3.72	25.76±6.95	26.96±5.79	0.75
Gender	13 women; 12 men	12 women; 13 men	13 women; 12 men	14 women; 11 men	___
Weight (kg)*	67.22±10.62	70.22±15.71	64.12±10.76	67.54±9.68	0.36
Height (m)*	1.70±0.08	1.73±0.09	1.69±0.08	1.69±0.07	0.23
Evaluated shoulder	10 dominant; 15 non-dominant	10 dominant; 15 non-dominant	13 dominant; 12 non-dominant	17 dominant; 8 non-dominant	___
Duration of pain (months)*	____	41.28±37.28	______	49.12±86.92	0.68

*Values are mean±standard deviation.

### Intrarater reliability

ICC and SEM values for intrarater reliability ranged from 0.95-0.97 and 0.30-0.42 cm, respectively, for both groups (Table 2).

**Table 2.** Intrarater and interrater reliability for assessing the pectoralis minor length with the tape measure in asymptomatic and symptomatic individuals.

	Trial 1* ^†^	Trial 2* ^†^	**ICC_3,1_ (95%CI)**	SEM *
Asymptomatic group (n=25)				
Rater 1	16.42±1.46	16.17±1.42	0.96 (0.92-0.98)	0.32
Rater 2	16.44±1.42	16.38±1.47	0.95 (0.90-0.98)	0.30
**Symptomatic group (n=25)**				
Rater 1	16.74±1.65	16.58±1.65	0.97 (0.93-0.98)	0.31
Rater 2	17.12±1.86	16.81±1.77	0.95 (0.91-0.98)	0.42
	Rater 1* ^†^	Rater 2* ^†^	**ICC_3,2_ (95% CI)**	SEM*
**Asymptomatic group (n=25)**	16.30±1.43	16.42±1.43	0.86 (0.68-0.94)	0.70
**Symptomatic group (n=25)**	16.66±1.64	16.97±1.80	0.87 (0.70-0.94)	0.84

*All units are in centimeters. † Values are mean±standard deviation. ICC: Intraclass correlation coefficients; SEM: Standard error of measurement.

### Interrater reliability

Table 2 also shows the interrater reliability data for both groups. ICC values for asymptomatic and symptomatic groups were 0.86 and 0.87, respectively. SEM values for the asymptomatic and symptomatic groups were 0.70 and 0.84 cm, respectively.

### Between-day reliability

Table 3 reports the between-day reliability data. ICC values for both groups were 0.95 SEM values for the asymptomatic and symptomatic groups were 0.40 and 0.41 cm, respectively. MDC values for the asymptomatic and symptomatic groups were 1.13 and 1.14 cm, respectively.

**Table 3.** Between-day reliability for assessing the pectoralis minor length with the tape measure in asymptomatic and symptomatic individuals.

	Day 1*^†^	Day 2* ^†^	**ICC_3,2_ (95% CI)**	SEM*	**MDC_95_** *
Asymptomatic group (n=25)	15.85±1.23	16.06±1.38	0.95 (0.90-0.98)	0.40	1.13
**Symptomatic group (n=25)**	16.23±1.55	16.27±1.68	0.95 (0.89-0.98)	0.41	1.14

*All units are in centimeters. † Values are mean±standard deviation. ICC: Intraclass correlation coefficients; SEM: Standard error of measurement; MDC: Minimal detectable change.

**Figure 2.**
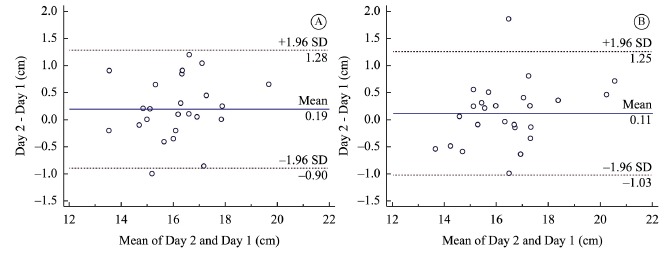
Bland-Altman plots for between-day agreement of measuring pectoralis minor length with a tape measure in asymptomatic (A) and symptomatic (B) groups.

Figure 2 presents the Bland-Altman plots for asymptomatic and symptomatic groups. Visual inspection of the plots for between-day reliability revealed that all mean differences were close to zero. No systematic biases were observed. The plots show a random scatter of points above and below the mean difference line, thus showing good agreement.

## Discussion

These results are similar to the measurement reliability estimates reported when using a caliper to assess PMI^10^. The current study adds pectoralis minor resting length and is, to our knowledge, the first study to report the between-day reliability of this measurement. Our results suggest that the tape measure method demonstrates very good reliability for a single rater and for different raters to measure the length of this muscle within the same day. Importantly, the study also provides estimates that this measurement has very good reliability for assessing pectoralis minor resting length over a seven-day interval. As one of the purposes for any clinical measure is to reliably quantify variables over time, the between-day reliability estimates are both practical and valugfable to clinicians. The stability of these measurements over seven days in the absence of an intervention provides clinicians with a way to document a real change in PM length when assessing for treatment effects.

Pectoralis minor muscle resting length can also be assessed by measuring the linear distance from the treatment table to the posterior aspect of the acromion with the patient in the supine position, as proposed by Kendall and Provance^4^. This method was later suggested to be useful to determine shortening of the pectoralis minor^5^. The reliability of this measurement was evaluated in subjects with and without shoulder symptoms, and excellent clinical intrarater reliability (ICC=0.92-0.97) was estimated^23^. It is important to note that this is an indirect measurement of pectoralis minor muscle length that demonstrated poor correlation with the PMI^3^. The PMI is calculated by dividing pectoralis minor resting length by subject height and multiplying by 100^1^. The poor correlation between these measurements^3^ likely reflects the fact that the table-to-acromion measurement is an indirect estimate of pectoralis minor length that can be influenced by scapula position changes from table and thorax forces and by altered body orientation to gravity. Conversely, the PMI uses the distance between the origin and insertion of the muscle in its calculation. Using this direct estimate between landmarks is advantageous when relying on a measurement to make treatment decisions within an individual patient.

Very good and good intrarater reliability was demonstrated when calculating the PMI in individuals with and without shoulder pain, respectively^10^. However, PM length was measured in the supine position in the previous study. The supine position is prone to misrepresenting true PM length for several reasons. First, the effect of gravity on the shoulder complex is changed, modifying the typical forces acting on the shoulder complex. Second, the plinth and weight of the thorax modify scapular position and PM length. In addition, typical functional activities of the upper extremity are done in standing, not supine. Finally, it has been shown that in supine the PM length measurement is influenced by the position of the upper extremity,^3^ with full internal rotation (palm down as used in Struyf et al.^10^) resulting in higher length estimates than either neutral or full external rotation. We contend that, in standing, the normal and constant influences on the shoulder (e.g. gravity or posture) are accounted for in the measurement and therefore make it more practical, functional, and reflective of the patient's true condition.

One may also argue if scapula dyskinesis could influence the results of the present study. We believe that dyskinesis would only interfere with the measurement if there was a dynamic component to the measure. Because the measurement is taken only in resting position, this is not a factor that influenced the study results.

As stated before, the PMI is not currently useful in clinical practice because normative values have not yet been established in the literature. Estimating PMI for each patient, based on a group of similar subjects, is not feasible to clinicians. As such, information about the direct muscle length may be more applicable in the clinical practice to identify individual subject changes after intervention.

The values for intrarater reliability observed for both symptomatic and asymptomatic groups were similar in the present study. In contrast, Struyf et al.^10^ reported higher intrarater reliability estimates in their group of symptomatic subjects. They explained these results as a learning effect of the examiners and due to the low variability of healthy controls. However, the asymptomatic group in their study was younger (~20 years) than the symptomatic group (~50 years), and the confidence intervals for ICC values were not provided, limiting full interpretation of the data. Our symptomatic group estimates also demonstrate that pain does not negatively influence the between day reliability of pectoralis minor muscle length because of the very similar results found in both groups. However, it is important to note that the mean duration of symptoms is quite long for this sample and cannot be generalized to individuals with acute pain.

Interrater reliability showed wide confidence intervals for both groups (0.68-0.94), despite the very good ICC and low SEM values that represent small variability of the measure. The sample size could have contributed to these wide confidence intervals, which lead to uncertainty about the point estimate, with the true reliability potentially being anywhere within the confidence interval. Consequently, clinicians should be cautious when interpreting pectoralis minor muscle length measured by more than one rater.

Our between-day measures showed a very good reliability over time (ICC=0.95), with a small variability (CI=0.89-0.98) in both groups. Regarding visual inspection of Bland-Altman plots, good agreement can be observed, reflecting the consistency in measuring pectoralis minor length using a tape measure on different days. The plots suggest a slightly greater length measurement on day 2 as compared with day 1, but the distribution of the difference scores indicates that there is no systematic bias. This information is important to clinical practice, because it shows stability of the measure in subjects with and without signs of impingement, and suggests that time does not influence the muscle length when no intervention is done.

Furthermore, the between-day MDC calculations suggest that a muscle length change greater than 1 cm is needed to identify a real change in pectoralis minor length on the same day and over time. However, a resting length change greater than 1 cm may not be possible given that the largest alteration in muscle length during passive pectoralis minor stretching was 0.77 cm, when performed in a similar position of the present study, with subjects in a sitting position without scapular stabilization^31^. It is not currently known how much muscle length change is possible or is associated with shoulder pathology, which makes interpreting the MDC a challenge. However, because the subjects in the present study were not evaluated for pectoralis minor shortening, our calculated MDC may not be directly applicable. It is possible that the MDC for those with a relatively shorter pectoralis minor length will be smaller because mean length and variability estimates may also be reduced. Similarly, individuals who demonstrate adaptive muscle shortening may also have more potential for an increase in muscle length than those without adaptive shortening. Moreover, variability in anthropometric characteristics may lead to differing MDC values so these results should be used with caution and applied only to individuals similar to the population used in this study. Additionally, studies that determine the minimal clinically important difference (MCID) for pectoralis minor length are needed to establish the amount of change that is meaningful and beneficial for the health status of the patient.

As the present study was conducted using only young subjects, our results cannot be generalized to older individuals or to those with other shoulder conditions.

## Conclusion

This study provides additional information about intra- and interrater reliability and important new knowledge of between-day reliability using a tape measure to assess pectoralis minor resting length in asymptomatic individuals and in individuals with signs of shoulder impingement. A single rater or different raters can reliably measure pectoralis minor within the same day, and a single rater can reliably use the measurement over a seven-day interval.
